# Optimized fault-tolerant data processing module for high-reliability CNN accelerator

**DOI:** 10.1371/journal.pone.0337338

**Published:** 2026-02-27

**Authors:** Sung-Kwang Yoon, Seung-Han Lee, Juhyeong Jo, Young-woo Lee

**Affiliations:** 1 Department of Electrical and Computer Engineering, Inha University, Incheon, South Korea; 2 Program in Semiconductor Convergence, Inha University, Incheon, Korea; 3 Department of Electrical and Electronic Engineering, Inha University, Incheon, South Korea; Chunghwa Telecom Co. Ltd., TAIWAN

## Abstract

Convolutional neural networks have become the foundation of image-inference tasks, with systolic array architectures providing enhanced computational performance and efficiency. However, the numerous processing elements (PEs) involved introduces significant challenges in terms of hardware overhead and reliability, which typically exhibit a trade-off relationship. Enhancing the efficiency and reliability of individual PEs can effectively address these challenges and substantially improve the overall performance of systolic array systems. We propose a module that can be implemented in PEs by integrating local binary patterns and min-max operations to reduce both power consumption and hardware size. This approach reutilizes the optimized architecture for fault detection, thus effectively minimizing the testing overhead. Our method enhances the overall system reliability by implementing a fault-PE bypass mechanism, thereby ensuring a robust operation. Experimental results show that the proposed module reduces the hardware area by 29.03% compared with previous circuits when synthesized with the Nan Gate 45 nm library. Furthermore, its dynamic power consumption is 13.72% lower compared with that of existing circuits when implemented on a field-programmable gate array. The results of a fault-injection experiment show that the proposed module reduces errors by up to 33.57% compared with previous circuits and that its test coverage exceeds 94%, with stuck-at 1 faults on PE input registers.

## Introduction

Convolutional Neural Networks (CNNs) are a type of Deep Neural Network (DNN) specifically designed for structured grid data, such as images. By employing convolutional layers, CNNs extract hierarchical spatial features, enabling efficient pattern recognition while reducing the reliance on manual feature extraction. CNNs offer several advantages, including local connectivity, weight sharing, and hierarchical feature representation, which significantly improve their performance in image classification, object detection, and segmentation tasks. Given these advantages, CNNs have become the foundation of numerous computer vision applications, including facial recognition, medical image analysis, and autonomous driving [1–5]. As their importance increases, researchers are increasingly focusing on maximizing CNN performance through both software optimization and the development of dedicated hardware accelerators [6–9]. Although algorithms based on graphics processing units (GPUs), which are optimized for parallel processing, dominate commercially available solutions, custom hardware accelerators leveraging field-programmable gate arrays (FPGAs) and application-specific integrated circuits (ASICs) deliver enhanced performance and improved energy efficiency [10–12]. Hardware accelerators, which are commonly known as artificial intelligence (AI) accelerators, have garnered considerable attention in recent years, particularly for balancing high performance and low power consumption in specific applications.

**Fig 1:**
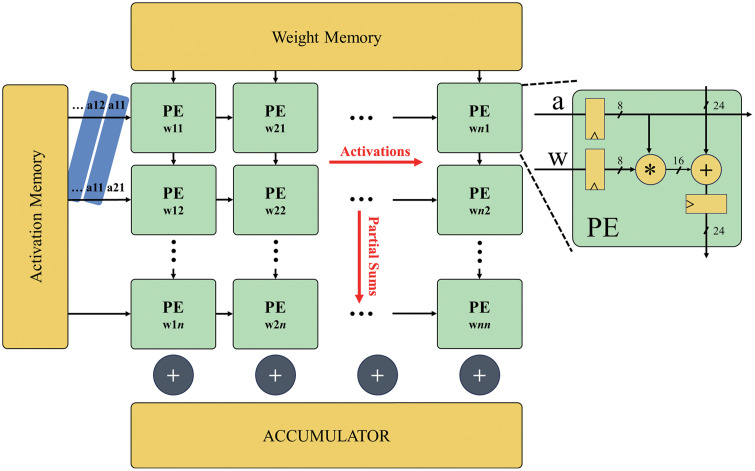
Typical structure of artificial intelligence accelerator with systolic-based array.

Systolic array architectures are widely used for efficient convolution operations in CNN accelerators. As shown in Fig 1, a typical CNN accelerator employing a systolic array comprises multiple processing elements (PEs) optimized for concurrent, simple computations. Google’s tensor processing unit is a standard implementation that utilizes a 256 × 256 PE systolic array to deliver high performance in large-scale data processing [13]. Because of the numerous PEs typically present in systolic arrays, even slight enhancements to the efficiency of individual PEs can substantially improve the overall system performance.

However, faults occurring within CNN accelerators, including those based on systolic arrays, can significantly disrupt the computation process. Dorostkar *et al.* [14] conducted a fault injection study on PIM-based CNN accelerators and demonstrated that faults in weight storage expose critical hardware vulnerabilities, with the early convolution layers and Batch Normalization layers identified as primary points of weakness. In addition, studies on systolic-array–based DNN accelerators have shown that faults injected into activation data can lead to accuracy degradation of up to 37.26% [15], indicating that activation data are also highly susceptible to transient errors. Furthermore, the Architectural Vulnerability Factor (AVF) of systolic arrays has been reported to reach as high as 34.8%, depending on the array size and dataflow configuration [16]. These findings collectively underscore the importance of considering hardware vulnerability alongside architectural optimization in the design of CNN accelerators.

Many researchers have attempted to optimize the power efficiency of systolic arrays to enhance their performance. Scale-out systolic arrays [17] introduce multiple “pods” to create scalable DNN inference accelerators. This architecture leverages a butterfly network to deliver high bandwidths and low latencies, which affords 1.5 × greater energy efficiency. From another perspective, ONE-SA [18] utilizes capped piecewise linearization to approximate nonlinear computations, which results in up to 25.73 ×, 5.21 ×, and 1.54 × higher computational efficiencies compared with those afforded by CPUs, GPUs, and SoCs, respectively. Additionally, enhancements to systolic array components, such as replacing carry propagation adders with carry save adders in MAC blocks, have demonstrated significant benefits. These modifications, as reported by Inayat et al. [19], yielded power reduction, area improvement, and delay reduction by up to 57%, 32%, and 6%, respectively.

The built-in self-test (BIST) is a widely adopted technique for self-diagnosis and recovery. STRAIT [20] implements a hybrid BIST approach that utilizes existing data paths and achieves 100% fault coverage with minimal power overhead. However, this results in a 5.2% increase in the total design area and an additional 8.7% increase in power consumption. Another approach integrates MAC units with a test circuit using an IEEE Std 1687 network, which achieves a diagnostic accuracy of 99.1% [21]. Similarly, RunSAFER [22] enhances reliability by monitoring errors in both data and control paths during runtime, thus resulting in a 12% improvement in power efficiency compared with conventional methods.

By extending the scope of previous studies, this study emphasizes the integration of CNN algorithms into a unified hardware platform to enhance the overall hardware efficiency. The proposed module selectively integrates key components of the CNN algorithm into a unified hardware module, thereby ensuring a streamlined and efficient design. Furthermore, by incorporating an optimized test circuit, the proposed module enhances its own reliability as well as that of the PEs. By applying this module to systolic array-based CNN accelerators, the design enhances power efficiency and significantly improves PE fault tolerance, thus resulting in greater overall system reliability. Based on experimental validation, this study demonstrates the effectiveness of the proposed methodology in achieving a low-power, high-reliability module for PEs in systolic array-based CNN accelerators.

Reliability, ensuring that the circuit functions correctly without errors, is just as crucial as hardware performance. Because of the considerable number of PEs within CNN accelerators, each one demands a particularly high level of reliability. The reliability of hardware architecture is generally ensured during the production phase through testing with Automatic Test Equipment (ATE). In addition, for systems demanding higher reliability, such as CNN accelerators used in autonomous vehicles, which are exposed to external environments or unexpected errors, built-in self-test (BIST) modules are commonly used. These self-test circuits consume additional power and area, potentially degrading hardware performance. Moreover, since the testing and operation phases are separated, it becomes difficult to promptly respond to faults that occur during normal operation, making it challenging to achieve high reliability. To address this limitation, techniques that enable ongoing testing are required. In such systems, however, an increase in test time can lead to operational delays and impose significant constraints.

To minimize trade-offs and enhance hardware performance while ensuring high reliability, this paper proposes a unified hardware module for PE that integrates multiple CNN layers, including LBP and portions of the BN operation, thereby significantly reducing the overall hardware overhead of the AI accelerator. While conventional systolic array structures focus mainly on optimizing fully connected and convolution layers, our approach extends optimization to include LBP and BN layers, enabling more efficient resource utilization. The resources saved through this integration are leveraged by reusing circuits to implement self-test modules for PEs, minimizing performance degradation due to additional reliability circuitry. Furthermore, the proposed test module which applies bypass circuits to test the PEs, enables real-time testing during accelerator operation. Unlike conventional approaches that conduct testing only during the idle state of the systolic array, the proposed method allows the PEs to perform real-time fault detection and handling during active operation, significantly enhancing fault coverage and ensuring robust reliability even under runtime conditions. By applying these optimized test circuits and background testing mechanisms, proposed methods achieve high fault tolerance in input registers with reduced AVF per PE, while maintaining uninterrupted operation through seamless fault management via PE bypass. This method enhances the efficiency of embedded AI systems and ensures their robustness even under fault-prone operating conditions.

## Backgrounds and related works

### Local Binary Patterns (LBPs)

**Fig 2:**
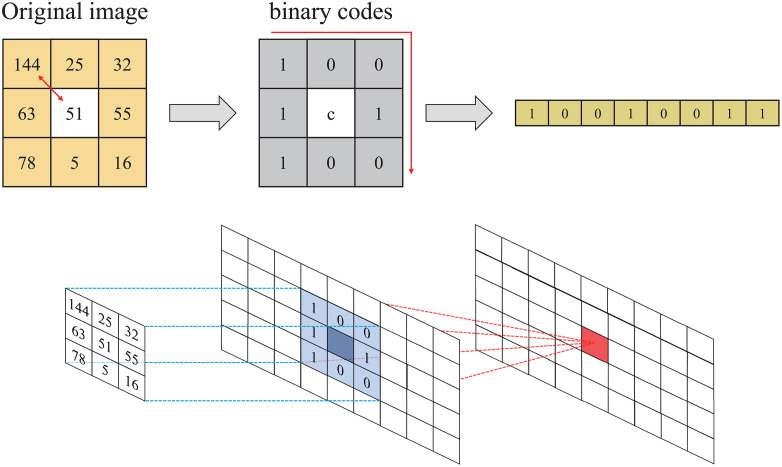
Example of pattern generation in local binary pattern algorithm.

LBPs are a commonly used data-preprocessing technique in image processing and computer vision, particularly for feature-extraction tasks, such as texture classification, face recognition, and feature detection [23]. In the context of CNNs, LBPs can be integrated as an additional layer during both training and inference to improve feature-extraction efficiency and reduce computational complexity. In the LBP method, the intensity of a central pixel is compared with that of its surrounding pixels in a specified region of an image, thus resulting in 8-bit binary patterns from a 3 × 3 pixel grid (Fig 2). Owing to its simplicity, LBPs enable rapid computations, thus rendering them highly suitable for real-time systems. When applied to FPGA-based real-time AI accelerators for CNN computations and inference, LBPs reduce data-processing loads, minimize hardware size, and decrease power consumption [24].

### Batch Normalization (BN)

BN is an algorithm used in various AI accelerators and can accelerate a process through stabilized training. Additionally, it reduces the changes in the input-data distribution for each layer during training. In BN, the activation values of each convolutional layer are normalized, thus effectively reducing the computational complexity and sensitivity to weight initialization while accelerating the training process and enhancing the inference accuracy [25]. Furthermore, BN addresses the internal covariate-shift problem by maintaining consistent input distributions across layers. This stability enables the use of higher learning rates and accelerates convergence during training, which is particularly beneficial for deep networks. During network training, the activation values of a batch are normalized. The mean and variance are calculated simultaneously across multiple samples, and this process is inherently dependent on the batch size. Larger batch sizes result in more stable normalization but at the cost of increased computational load and higher complexity. Despite this increase in computational demand, applying BN significantly enhances the overall accuracy of CNN models, thus rendering it a critical component in modern deep-learning architectures [26].

### Hardware-optimized BN

BN enhances training stability, accelerates convergence, and improves model accuracy in deep learning. However, the computation of the mini-batch mean and variance introduces additional data complexity, thus increasing power consumption and the memory-access overhead. RBN [27] eliminates the necessity for precise mini-batch variance calculations via the following equation:


$$\begin{array}{c} \tilde{\sigma }=\text{C}\cdot \frac{\sum_{k=\text{1}}^{n}\text{max}\left(x_{j}^{k}\right)-\sum_{k=\text{1}}^{n}\text{mi}n\left(x_{j}^{k}\right)}{\text{2}\cdot n} \end{array}$$ (1)

Here, $x_{j}^{k}$ denotes the jth element in the kth feature of a mini-batch; n represents the batch size; and C is defined as $\text{1}/\sqrt{\text{2}\text{ln}(m)}$, where m is the mini-batch size. RBN approximates the standard deviation to reduce the computational load, thereby accelerating the training process. Therefore, RBN is essential for implementing a BN layer in the hardware.

### Low-power digital comparators

Both LBPs and min-max operations can be efficiently implemented using digital comparators. Various optimization techniques have been investigated to enhance the efficiency of low-power digital comparators. The bitwise competition logic comparator proposed by Kim & Yoo eliminates arithmetic operations [28], thereby reducing power consumption and hardware area compared with conventional methods. The parallel prefix tree comparator introduced by Saleh Abdel-Hafeez et al. [29] minimizes dynamic power consumption by reducing the switching activity but increases the transistor count, thus rendering it less suitable for low-power applications. Recent studies have focused on EX-OR-NOR gate-based low-power comparators [30], which offer superior power efficiency and delay performance. This type of digital comparator is highly suitable for compact, low-power designs and can be effectively integrated into CNN accelerators.

### Permanent faults in PE registers

During the operation of a CMOS-based CNN accelerator, transient faults occur far more frequently than permanent faults. However, such transient faults generally do not have a significant impact on the overall inference results, as their effects do not persist or propagate continuously to subsequent PEs [31]. In contrast, permanent faults in the register cells of individual PE can severely affect the inference accuracy. Such faults in input registers can result in significant computational errors. Jeff (Jun) Zhang et al. [32] conducted an experiment to examine the effect of permanent faults in PEs on the inference accuracy of AI accelerators. They introduced stuck-at faults into the internal nodes of a gate-level netlist to simulate such failures. The results indicated that the accuracy decreased significantly from approximately 95% to 65% with only eight faulty PEs. This shows that a small number of faults can substantially degrade the accuracy. However, this problem can be solved by bypassing faulty PEs because the calculation error is more critical for accuracy than the PE loss. FAP + T, which manages timing errors between PEs with bypass circuits, exhibits only an 8% decrease in accuracy with a 50% PE fault rate [33].

### Proposed module architecture

#### Hardware integration of LBP and BN.

Integrating an optimized BN algorithm with LBPs in hardware can significantly enhance the overall performance of CNN accelerators. However, implementing these layers as separate hardware modules increases power consumption and the silicon area, thus rendering the approach cost intensive. By integrating the hardware of LBPs and BN into a unified module, the associated overhead of separate processing units can be minimized, thereby enhancing both efficiency and resource utilization. When designing the BN module using RBN, a comparator-based min–max circuit is utilized to approximate the mean and standard deviation. Similarly, the LBP employs a comparison circuit to evaluate the central pixel against its surrounding pixels. By leveraging this shared computational framework, both the layers can be efficiently integrated and optimized within a single hardware module.

#### Proposed module.

**Fig 3:**
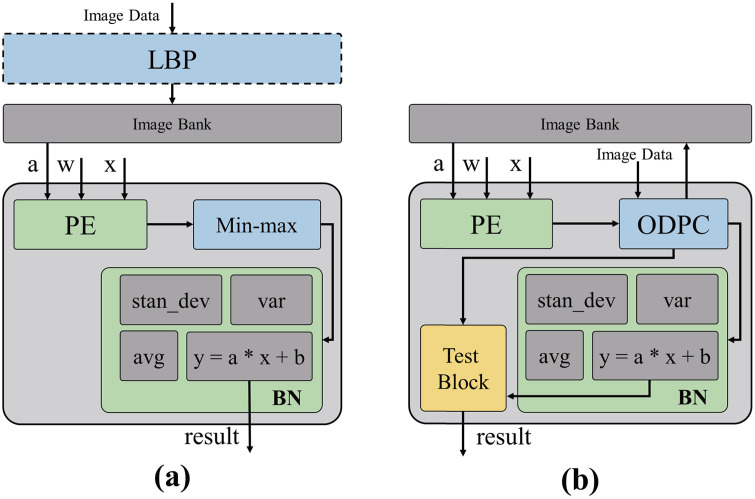
Block diagram of previous and proposed PE designs. (a) previous LBP and min–max circuits; (b) proposed PE with optimized data-processing circuits and test block.

The conventional comparator architecture implemented for LBPs in an FPGA employs a subtractor-based comparison approach [34], which results in high power consumption and reduced processing speed. The increased computational complexity and processing delay caused by carry or borrow propagation in the subtractor contribute primarily to this issue. Due to the inherent characteristics of a subtractor, eliminating borrow propagation is infeasible, even with optimal optimization [35]. Therefore, a more efficient circuit designed specifically for comparison is required. To overcome these limitations, we developed an optimized data-processing circuit (ODPC) to replace the LBPs and min–max circuits shown in Fig 3(a). The ODPC is an XOR gate-based comparator that performs bit-wise computations to reduce power consumption. The proposed circuit, deviating from conventional subtract-based designs, performs comparison operations using a single gate level, thereby minimizing hardware area and reducing power consumption. Furthermore, ODPC introduces a additional optimization strategy by unifying the LBP and BN layers into a single hardware structure, achieving efficiency not only at the circuit level but also through algorithm-level integration. These design advantages contribute to superior resource utilization, positioning the proposed module as a highly effective solution for embedded AI systems.

Because the ODPC is designed to perform both LBPs and min-max operations, its data-processing complexity is greater than that of the conventional min–max circuit. This complexity increases the ODPC’s sensitivity to faults, thus potentially compromising the system reliability. The test block in Fig 3(b), which is designed specifically for the ODPC, strengthens fault tolerance while maintaining reduced hardware footprint and low power consumption compared with previous circuits, thus significantly enhancing the overall system reliability.

The proposed module is incorporated into each PE. During the LBP phase, the ODPC processes 8-bit image data and stores the resulting LBP output in an image bank. During inference, the ODPC operates as part of the BN processor, where min-max operations are executed on the 16-bit outputs of the PEs. These min-max results are used to approximate the mean and variance in the RBN algorithm. During the test operation, the test block verifies the reliability of the ODPC while simultaneously evaluating the integrity of two input registers within the PE which is image data register and weight resister. By temporarily pruning the PEs undergoing testing with a bypass circuit in the test block, the latency overhead during inference is eliminated. Additionally, pruning is maintained upon fault detection; therefore, permanent faults remain continuously bypassed, while transient faults are released and restored during the subsequent test sequence. This approach prevents the detected faults from affecting the overall accuracy of the inference process effectively. By incorporating this simple module into the PEs, the primary computations of the LBP and the min-max operations of RBN can be efficiently replaced with a low-power alternative, thus facilitating the implementation of a compact and energy-efficient PE. Moreover, the reliability of the proposed module is verified by detecting and managing faults in systolic array during the operation. This online test structure extends the conventional fault coverage area by detecting faults that occur during the operation.

#### ODPC.

The ODPC is designed to consume less power and hardware area with optimizing comparing circuits. Furthermore, unlike other AI accelerator designs, the proposed method achieved further optimization by integrating LBPs and min-max comparison operations into a unified module. The circuit is implemented to perform bitwise computations using XOR gates. The ODPC utilizes eight XOR gates to compare 16-bit input values bit by bit, thus resulting in an 8-bit output. Based on this output, the priority encoder identifies the first differing bit between two sets of input data. By verifying the first differing bit position in the original input data, a value of “0” in the input data indicates that the data are smaller, whereas a value of “1” indicates the data are larger than the comparison data. This circuit effectively allows the comparison of 8-bit or larger data sizes. The ODPC employs these operation circuits in parallel, thus enabling two values to be processed per cycle in the LBP mode and the “min” and “max” values to be computed in the min-max mode simultaneously. Through this approach, we secured additional hardware resources for the test modules described later and maximized the benefit by reusing the corresponding circuits during test operations. The following section provides a detailed explanation of the two ODPC modes, i.e., the LBP and min-max modes.

#### LBP mode.

**Fig 4:**
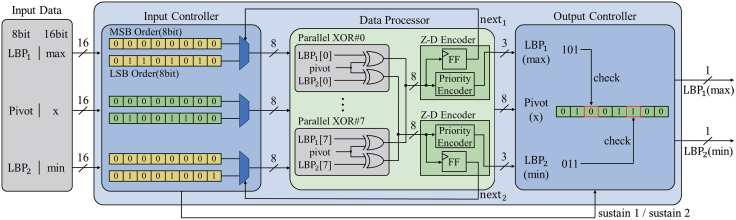
Block diagram of proposed optimized data-processing circuits.

In the LBP mode, the pivot, which represents the value of the central pixel, and the surrounding pixels, LBP1 and LBP2, are provided as 8-bit values. An example of an LBP operation is shown in Fig 4. In this instance, LBP1 begins to differ from the pivot at the third bit from the MSB-order. When this 16-bit data passes through the eight parallel XOR gates, it yields an output value of “00100110,” which is subsequently sent to the Z-D encoder. Inside the Z-D encoder, the priority encoder identifies the third bit from the MSB-order as the first “1” in the data and sends “101” to the output controller, which is the third bit. The output controller reads the bit value at position “101” from the pivot, which is “0,” which results in a final output of “1” from LBP1, thus indicating that LBP1 is larger than the pivot. In the case of LBP2, where the sixth bit differs, the corresponding value of “011” from the pivot is “1.” Therefore, the final output of LBP2 is “0,” thus indicating that LBP2 is smaller than the pivot. The ODPC in the LBP mode repeats this operation four times to generate 8-bit LBP data for a 3 × 3 pixel block.

#### Min-max mode.

The min-max mode operates similarly to the LBP mode but differs in two key aspects: it inverts the final output during min operations and introduces additional signals to support 16-bit computations. In the ODPC, the “next” signal is crucial in processing 16-bit data by segmenting them into 8-bit operations. Algorithm 1 describes the interaction between the input controller and data processor, where the “next” signal guides the transition between the MSB- and LSB-order operations. When the MSB-order values of the compared inputs match, the “next” signal prompts the input controller to process the LSB-order data. If the received LSB-order data are different, then the “next” signal is reset to “0.”

However, implementing this logic with a combinational design can result in a critical loop, thus causing the input controller to repeatedly perform unnecessary operations until a new input value is introduced. This looping behavior reduces the computational accuracy and overall system reliability. Hence, a clock-synchronized operation with flip-flops is included in the priority encoder, as described in Algorithm 2.

**Algorithm 1** Input Controller and Data Processor (Sequential)

1: Input

*x*: x value (16-bit)

*min*: min value (16-bit)

*max*: max value (16-bit)

2: **Output**

*next*: to output controller (1-bit)

result: priority-encoder result (3-bit)

3: **Initialize** set *next* to *0*

4: **for** (until a new value is received) **do**

5:  **if** (*next* == *0*) **then**

6:    send MSB-order 8-bit data to data processor

7:    **if** (XOR results are all 0) **then**

8:     set *next* to *1*

9:  **if** (*next* == *1*) **then**

10:   send LSB-order 8-bit data to data processor

11:   **if** (XOR results are not all *0*) **then**

12:    set *next* to *0*

13: **return** *next*, *result*

**Algorithm 2** Input Controller and Data Processor (Combinational)

1: **Input**

*x*: x value (16-bit)

*min*: min value (16-bit)

*max*: max value (16-bit)

*clk*: clock (1-bit)

2: **Output**

*next*: to output controller (1-bit)

result: priority-encoder result (3-bit)

3: **Initialize** set *next* to *0*

4: **for** (first *clk* rising) **do**

5:  **if** (*next* == *0*) **then**

6:    send MSB-order 8-bit data to data processor

7:    **if** (XOR results are all *0*) **then**

8:      set *next* to *1*

9: **for** (second *clk* rising) **do**

10:  **if** (*next* == 1) **then**

11:   send LSB-order 8-bit data to data processor

12:   **if** (XOR results are not all 0) **then**

13:     set *next* to 0

14: **return** *next*, *result*

The ODPC employs a “sustain” signal to retain prior values, thus facilitating parallel processing for 16-bit min-max operations. The input controller generates the “sustain” signal upon detecting distinct “next” signals and forwards it to the output controller. In scenarios where the max result is derived from the MSB-order but the min result requires computation in the LSB-order, the parallel circuits may erroneously propagate the LSB-order value of “x” to the max output, thus generating an incorrect result. The “sustain” signal addresses this issue by ensuring that the output controller retains the MSB-order result. Table 1 outlines the decision-making process of the input controller based on the states of the “next1” and “next2” signals, which correspond to the subsequent operations for min and max calculations, respectively.

**Table 1:** Input control decisions of the input controller.

Next1	Next2	Input Control Decision
0	0	Send MSB order
0	1	Send min LSB order and sustain 1
1	0	Send max LSB order and sustain 2
1	1	Send LSB order

### Test block

**Fig 5:**
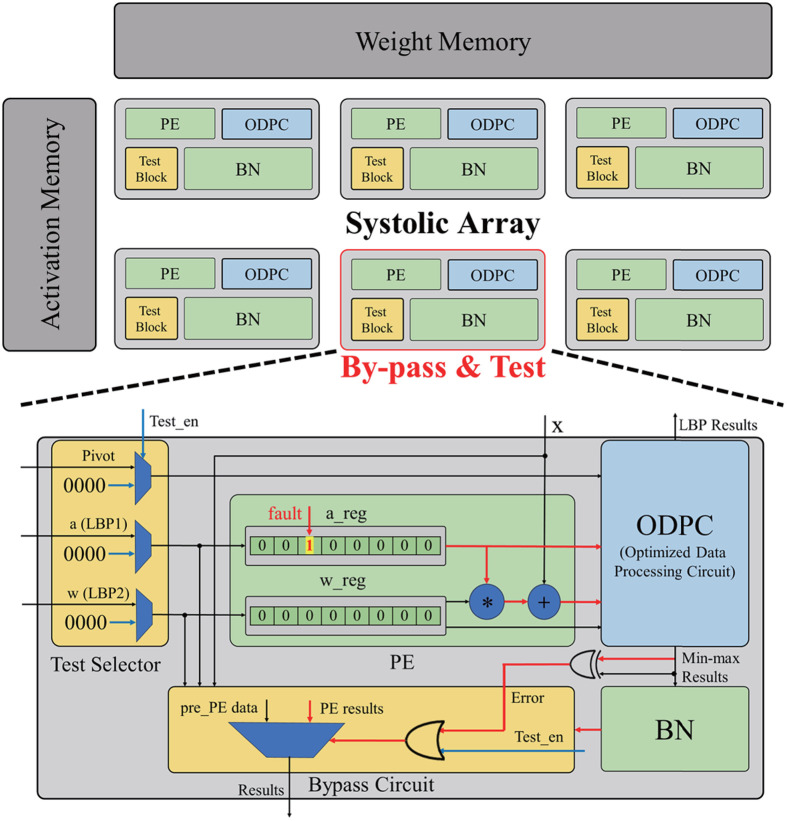
Block diagram of proposed module with data-processing circuit for error detection and test block.

Conventional test structures do not perform testing during the operation of the systolic array, making them ineffective in detecting faults that occur at runtime. To address this limitation, we propose a novel test block that enables testing during the active operation of the systolic array, thereby enhancing the reliability of embedded AI systems. The proposed test block comprises three primary components: the test selector, ODPC, and bypass circuit, as depicted in Fig 5. Under a normal operation without testing, the test selector transmits the input values without modification. When a test signal is received, all outputs are forced to zero, thus enabling the ODPC to detect faults in specific cells of the input registers. To reduce the hardware overhead, the ODPC is reused for fault detection by leveraging its capability to identify differences between input values. Under a stuck-at 1 fault, the ODPC detects discrepancies between two inputs, thus resulting in different values between two binary outputs. This fault can be efficiently identified using a single XOR gate. This approach enhances reliability by effectively identifying discrepancies caused by faults while minimizing the necessity for an additional hardware area. Despite the limitations of this circuit, which cannot detect stuck-at 0 faults, it demonstrates exceptional performance in terms of reduced power consumption and optimized hardware area. By leveraging this approach, the proposed module reduces the sensitivity to stuck-at faults while maximizing the advantages of compact area and low power consumption. The bypass circuit enhances the reliability of the CNN accelerator by facilitating PE pruning. It operates by directly transmitting the output of the preceding PE during self-testing, or by detecting the error signal from the ODPC with an XOR gate. As bypassing the PEs marginally affects the overall accuracy of CNN computations, the testing process proceeds without disrupting the primary operations. Additionally, once a fault is detected in the input registers of the PE, the bypass mechanism remains active until the fault is no longer detected, thus effectively isolating the faulty PE from the CNN inference process to prevent degradation to the model accuracy. The proposed module enables real-time self-testing through the bypass circuit while improving the fault tolerance of the CNN accelerator by pruning defective PEs.

## Experimental results

**Fig 6:**
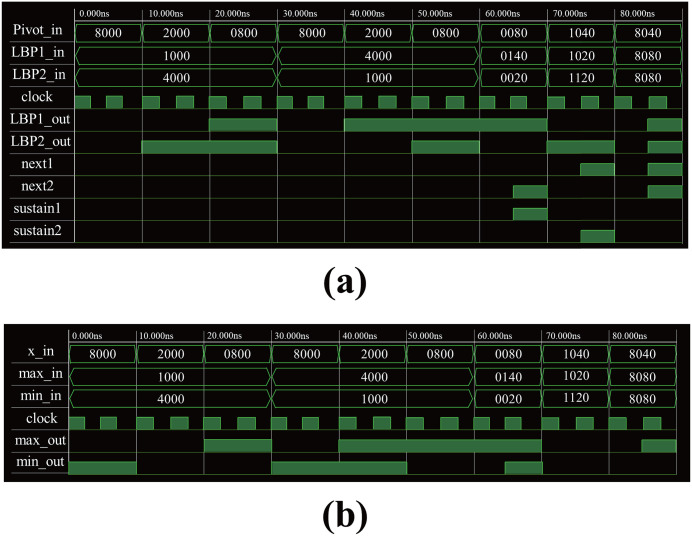
Test-bench results for functional validation of proposed data-processing circuit. (a) LBP mode; (b) min–max mode.

Simulations were conducted to verify the functional operation of the proposed module and evaluate the hardware size and dynamic power. For the experiments, we used Synopsys Design Vision for synthesis with the “Nan Gate 45 nm Open Cell Library” and implemented it on Xilinx’s Zynq Ultrascale +. Fig 6 shows the test-bench results for the functional validation of the ODPC in the proposed module. To prevent a critical loop with the “next” signal, the ODPC requires two clock cycles for a 16-bit computation. For the first input, the operation is completed within the first clock cycle. However, after 60 ns of inputs, a comparison of the pivot and LBP1 in the MSB order shows a transmission of the “sustain 1” signal at the second clock rising. This process preserves the value from the previous clock using the “sustain” signal. The operation of the min-max mode is illustrated in Fig 6(b). The “max” operation shows no change in the output data under the LBP mode, whereas in the “min” operation, “1” is output when the “min” value is smaller than the “x” value. Through this experiment, we confirmed that all computations required for both LBP and BN can be handled within a single module.

In AI embedded systems, hardware area plays a crucial role in determining overall system efficiency, particularly for space- and weight-constrained platforms such as drones and autonomous vehicles, where compactness directly impacts performance and applicability. However, the inherently complex and multi-layered structure of AI models require sophisticated hardware operations, often increasing the size of AI accelerators. The hardware size of the ODPC, as synthesized using the previously mentioned library, was measured to be 250.96 GE. This measurement was performed using an equivalent gate count based on a 2-input NAND gate with an area of 1.22 μm². A 29.03% reduction in hardware size was indicated compared with the results obtained using two separate, independent circuits, as shown in Table 2. Among the previous circuits, the LBP operation exhibited a relatively large area overhead, primarily because of the additional hardware required to control carry propagation and mitigate data overflow during the subtraction process. By contrast, the proposed approach leveraged bitwise operations using XOR gates to effectively reduce redundant hardware components. Consequently, the proposed method enables a more compact and efficient circuit compared with conventional LBP implementations.

**Table 2:** Hardware size measured at the equivalent gate count of the 2-input NAND gate.

Method	Circuits and Block	Total Area (GE)
Previous	LBP [34]	240.80
	Min-max [27]	158.86
	Total	399.55
Proposed	ODPC	250.69
	Test Block	33.03
	Total	283.56

**Fig 7:**
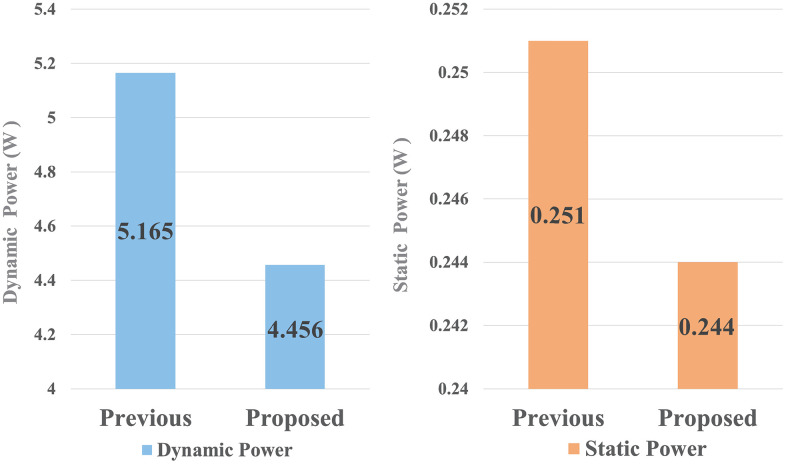
Power consumption of previous and proposed modules after FPGA implementation.

**Fig 8:**
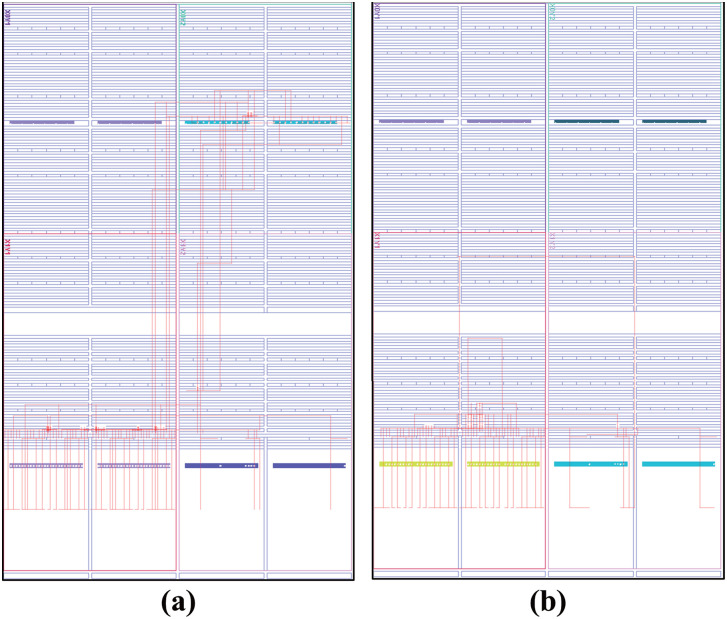
Design view of previous and proposed modules. (a) LBP and min–max circuits; (b) optimized data-processing circuit.

Power consumption presents a significant challenge in embedded AI systems, as AI inference requires additional energy beyond the baseline operational power. Fig 7 shows the power consumption of proposed module implemented on an FPGA. The results indicate that the dynamic power of the proposed module was 0.079 W lower than that of previous circuits. Furthermore, the static power consumption of our module was 0.007 W lower than that of previous circuits. These results demonstrate that applying this method to each PE in a 16 × 16 systolic array can reduce dynamic power consumption by up to 20.224W. Fig 8 shows a device view of the FPGA, where the LBP with min-max and the ODPC were implemented individually. Fig 8 shows that the ODPC operated under a simpler and shorter net configuration compared with previous circuits. Achieving both enhanced system reliability through the integration of additional test modules and reduced power consumption is highly challenging. The previous architecture demonstrates higher power consumption and lacks dedicated test modules, leading to inefficiencies and limited reliability assurance. In contrast, the results of experiments clearly show that the proposed module resulted in a smaller hardware area. And a lower dynamic power was achieved despite additional real-time self-test circuits implemented, thus presenting higher circuit reliability.

The fault-injection experiment was conducted to assess the reliability of the proposed module and to compare its performance with that of previous circuits. Stuck-at 0 and Stuck-at 1 faults were systematically introduced into each input bit in the synthesis design, and their effects were evaluated by comparing the resulting faulty outputs against the expected correct outputs via a test-bench simulation. Considering the distinct operational characteristics of the LBP and min-max functions, dedicated input configurations were designed for their respective evaluations. For the LBP operation, faults were injected into both LBP1 and LBP2, which generated 32 fault scenarios, with the input types classified based on the output states and random values selected to represent outputs of “00,” “01,” “10,” and “11.” Each category contained 10 randomly selected samples, which resulted in 40 unique input configurations, thus yielding 1,280 fault-injected scenarios. For the min-max operation, faults were injected into the 16-bit min and max inputs, which resulted in 64 fault scenarios. Additionally, the input types were classified into four categories: computations completed in the MSB order, cases requiring “next” and “sustain” signals, scenarios where min equals max, and general cases with fully randomized values. Each category included 10 random samples, thus yielding 40 distinct input types and culminating in 2,560 fault-injected scenarios.

Using the fault-injection experiment results, the architectural vulnerability factor (AVF) of the proposed module was calculated and compared with that of conventional circuits. The AVF, defined as the ratio of faults affecting the output to the total number of fault scenarios, was determined as follows:


$$\begin{array}{c} AVF(\%)=\left(\frac{Fault\;Affecting\;the\;Output}{Total\;Injected\;Faults}\right)\times \text{1}\text{0}\text{0} \end{array}$$ (2)

**Fig 9:**
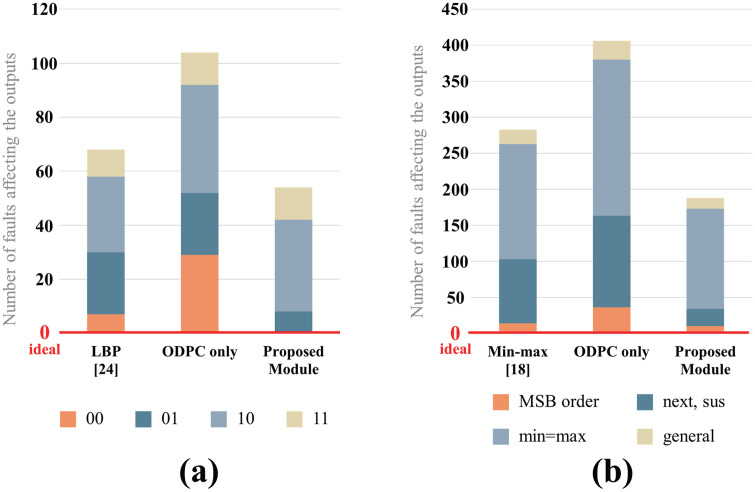
Results of fault-injection experiments. Number of faults affecting outputs in (a) LBP experiments and (b) min–max experiments.

The fault-injection experiments demonstrated that the ODPC exhibited greater susceptibility to faults than conventional circuits. This increased vulnerability stems from the ODPC’s reliance on additional signals to execute computations in 8-bit segments and its parallel structure, which shares comparison values. The proposed module resolves this issue by incorporating an optimized test block designed for the ODPC, thereby facilitating self-testing to effectively suppress fault-induced errors. Consequently, the number of errors reduced by 20.58% and 33.56% for the LBP and min-max methods, respectively. Fig 9(a) shows a comparison between the fault-injection experiment results with the results of the proposed method with LBPs. When the expected output was “00,” the testing mechanism successfully corrected all errors. This outcome can be attributed to the nature of stuck-at 0 faults, which do not affect “00” outputs. Therefore, addressing only stuck-at 1 faults was sufficient to eliminate errors.

**Fig 10:**
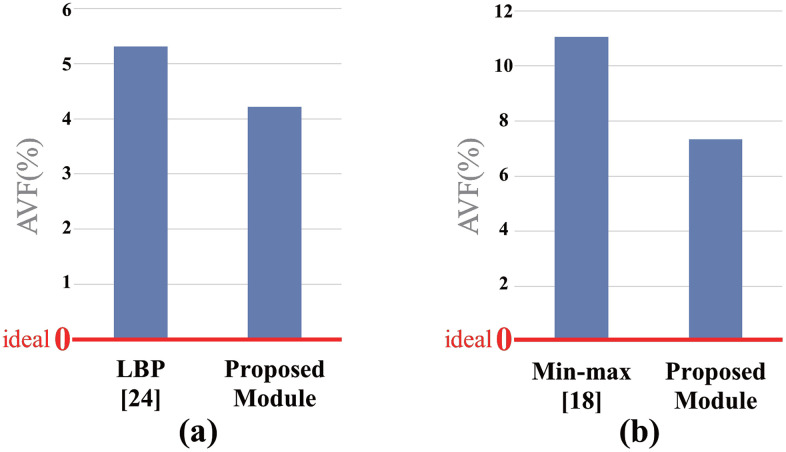
AFVs from fault-injection experiments. (a) LBP experiments; (b) min–max experiments.

Fig 10 shows a comparison of the AVFs calculated from the fault-injection experiment results. In the LBP and min-max experiments, the proposed module exhibited reduced AVFs by 33.57% and 20.59%, respectively, compared with conventional circuits.

Table 3 presents the fault simulation results for two 8-bit input registers within the PE. The proposed fault-detection mechanism identifies faults by comparing the outputs of the two registers and detecting discrepancies. However, when faults occur at the same position in both registers, they remain undetected. Such cases arise only when the number of faults is even, which accounts for a negligible portion of all fault scenarios. Consequently, when considering only stuck-at 1 faults, the proposed method maintained a test coverage exceeding 94% for the input registers within the PE. As the number of faults increased, the proportion of cases in which the proposed module fails to detect faults decreased, thus resulting in an overall increase in coverage. The proposed architecture achieves nearly 100 percent coverage for stuck at one faults in the real time test domain and this indicates an expansion of test coverage into areas that were previously untestable specifically during the operational phase of the systolic array.

**Table 3:** Test coverage of proposed module for PE input registers.

Number of Faults	1	2	3	4	5	6
Detected	16	128	816	3,848	15,504	54,114
Total Coverage	50%	46.71%	50%	49.57%	50%	49.86%
Stuck at 1 Coverage	100%	94.11%	100%	99.27%	100%	99.72%

## Conclusion

The systolic array, widely adopted in CNN accelerators, demands both optimization and enhanced reliability to meet the increasing performance requirements of embedded AI systems. This paper presents a high-reliability optimization module for PEs using the ODPC structure, which integrates LBP and BN layers in addition to conventional fully connected and convolution layers. By extending the design scope to include LBP and BN, the proposed module achieves reduced hardware area and low-power operation simultaneously. Moreover, the ODPC incorporates a real-time self-testing mechanism and a PE bypass structure that enables fault detection during active operation. Unlike conventional approaches, our method expands the test coverage by allowing continuous monitoring of faults during online execution. This architectural advantage is particularly effective for safety-critical applications such as autonomous vehicles, where uninterrupted, high-reliability AI acceleration is essential. Experimental results confirm the effectiveness of the proposed design: a 29.03% reduction in hardware area was achieved using a 45 nm Nan Gate library, and dynamic power consumption was lowered by 13.72% on Xilinx’s Zynq UltraScale +. Fault injection experiments further demonstrated a reduction in AVF by 33.57% and 20.59% in LBP and min-max operations, respectively. The proposed module also achieved over 94% test coverage for stuck-at-1 faults in PE input registers. These findings validate the proposed architecture as a compact, energy-efficient, and highly fault-tolerant solution for next-generation CNN accelerators for embedded AI systems.
